# Effect of Cataract Type and Severity on Visual Acuity and Contrast Sensitivity

**Published:** 2011-01

**Authors:** Javad Heravian Shandiz, Akbar Derakhshan, Ameneh Daneshyar, Abbas Azimi, Hadi Ostadi Moghaddam, Abbas Ali Yekta, Seyed Hosein Hoseini Yazdi, Habibollah Esmaily

**Affiliations:** 1 Department of Optometry, Mashhad University of Medical Sciences, Mashhad, Iran; 2 Eye Research Centre, Mashhad University of Medical Sciences, Mashhad, Iran; 3 Department of Optometry, Tehran University of Medical Sciences, Tehran, Iran; 4 Depatment of Biostatistics, Mashhad University of Medical Sciences, Mashhad, Iran

**Keywords:** Contrast Sensitivity, Glare Sensitivity, Visual Acuity, Age-Related Cataract

## Abstract

**Purpose:**

To determine the effect of cataract type and severity in eyes with pure types of age-related lens opacities on visual acuity (VA) and contrast sensitivity in the presence and absence of glare conditions.

**Methods:**

Sixty patients with senile cataracts aged 40 years or older with no other ocular pathologies were evaluated for VA and contrast sensitivity with and without glare. Lens opacities were classified according to the Lens Opacities Classification System (LOCS) III. VA was measured using the Snellen chart. Contrast sensitivity was measured with the Vector Vision CSV-1000E chart in the presence and absence of glare by calculating the area under log contrast sensitivity (log CS) function (AULCSF).

**Results:**

Cataracts were posterior subcapsular in 26 eyes, cortical in 19 eyes and nuclear in 15 eyes. VA significantly decreased with increasing cataract severity and there was significant loss of contrast sensitivity at all spatial frequencies with increasing cataract severity. AULCSF significantly decreased with increasing cataract severity in the presence and absence of glare conditions. Contrast sensitivity was significantly reduced at high spatial frequency (18 cpd) in cortical cataracts in the presence of glare in day light and at low spatial frequency (3 cpd) in night light.

**Conclusion:**

Increased cataract severity is strongly associated with a decrease in both VA and AULCSF. Contrast sensitivity scores may offer additional information over standard VA tests in patients with early age-related cataracts.

## INTRODUCTION

Visual acuity (VA) is the conventional and standard test of visual function in patients with cataracts. However, some patients retain relatively good VA, yet complain of poor vision.^1^–^3^ In these patients, other tests of visual function such as contrast sensitivity (CS) should be evaluated.^4^–^7^

Contrast sensitivity measures visual performance under real-life conditions and therefore can better quantify patients’ capabilities.^8^,^9^ Cataracts are thought to increase intraocular light scatter, which can decrease retinal image contrast and adversely affect contrast sensitivity^10^, which is affected far more than visual acuity in patients with cataracts.^1^,^10^,^11^ Fujikado et al^12^ found that higher-order aberrations are significantly correlated with contrast sensitivity at intermediate to higher spatial frequencies in eyes with cataracts.

Patients with cataracts often complain of glare, for example from bright sunlight or car headlights: some find this glare more disabling than a moderate drop in visual acuity. These symptoms have been termed as glare disability.^13^,^14^

It has been reported that glare disability with cataracts does not correlate with visual acuity.^15^–^17^ Therefore, a patient may have significant glare disability despite good visual acuity. Abrahamsson and Sjostrand^18^ found that reduced contrast sensitivity functions as a measure of glare disability in patients with early cataracts. They suggested that a normal individual has a glare score that is almost independent of luminance level, while patients with cataracts have a marked increase in glare sensitivity when luminance is decreased. Jaffe^19^ suggested that glare disability can be used as an adjunct to VA measurement in order to objectively assess the need for cataract surgery. There are some commercially available charts for measuring contrast sensitivity without glare source. It has been reported that a patient may have good visual acuity but significant contrast and glare disability.^20^–^24^ The aim of this study was to investigate the discriminative ability of the CSV-1000E test with glare source to measure contrast sensitivity and glare disability in patients with early cataracts.

## METHODS

Our patients were selected from participants of an ongoing descriptive study at Khatam-Al-Anbia Eye Hospital on the natural history of age-related cataracts. Patients aged 40 to 75 years with no other ocular diseases other than cataracts (glaucoma, optic nerve disease, macular diseases or anterior segment disease) were enrolled for the study. All subjects had at least one eye with a single type of cataract (pure cortical, pure nuclear or pure posterior subcapsular). Preference was given to the eye with the highest lens opacity grade and to the right eye when both eyes had similar degrees of lens opacities.

**Figure 1:**
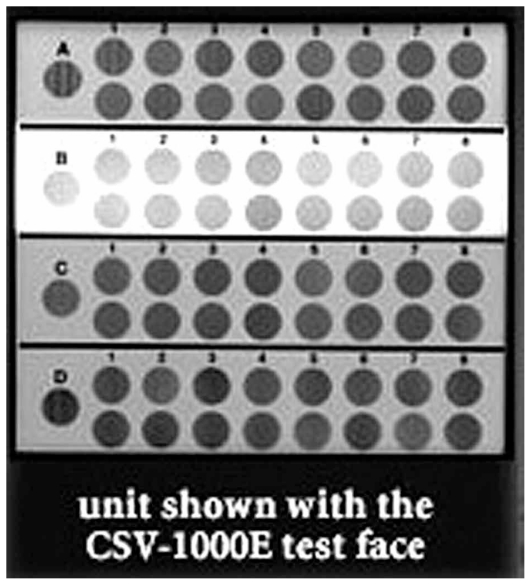
Contrast sensitivity test system.

Each patient underwent a complete ophthalmologic and optometric eye examination. Best corrected VA (BCVA) was determined monocularly using the Snellen chart and contrast sensitivity was measured with a sine-wave grating at spatial frequencies of 3, 6, 12, and 18 cpd using the Vector Vision CSV-1000E chart (Vector Vision, Haag-Streit, Harlow, UK). Glare sensitivity was measured in day and night light. For this purpose, the Vector Vision CSV-1000E contrast test houses a series of photocells that monitor and calibrate the instrument light level to day or night light (Fig. 1). The chart is back-illuminated and viewed from 8 feet with mean luminance of 85 cd/m^2^ (low photopic condition).^24^ The chart consists of four rows of patches. Each row presents 17 circular patches 1.5 inches in diameter. The first patch on the far left in each row presents a very high contrast grating (sample patch). The remaining 16 patches appear in 8 columns across the row. In each column, one patch presents a grating and the other patch is blank. The patches that present grating decrease in contrast moving from left to right across the row. The patient indicates whether grating appears in the top patch or the bottom patch for each column.^25^

Sensitivity values were transformed into a logarithmic scale, and each subject’s area under the log contrast sensitivity (log CS) function (AULCSF) was calculated. AULCSF represents sensitivity of the entire visual system to contrast and is calculated as follows:^24^

$$AULCSF=0.5\;\text{log}\;CS(3\frac{c}{d})\text{log}\;CS(6\frac{c}{d})+0.75\;\text{log}\;CS(12\frac{c}{d})+0.25\;\text{log}\;CS(18\frac{c}{d})$$

Classification and grading of lens opacities was performed using a slitlamp and according to the Lens Opacities Classification System III (LOCS III). The grade for each feature was derived by locating the image of the patient’s lens on the scale of severity for each feature represented in the colour transparency.^25^

All statistical analyses were performed only for the eye with pure age-related cataract. For statistical analysis, Snellen acuities were converted to equivalent values of visual angle using the decimal scale. Descriptive and inferential data analyses were performed using SPSS, version 11.5. Correlation was tested between cataract severity and age using a general linear model to control for the effect of age and sex. Repeated measurement of analysis of variance (ANOVA) was performed to test the difference between contrast sensitivity with and without glare at all spatial frequencies for each type of cataract. Significant differences between each paired group were then evaluated by the least significant difference LSD test.

## RESULTS

Sixty eyes of 60 patients with mean age of 59.0±9.6 (range, 40 to 75) years were studied. Cataracts were pure posterior subcapsular (PSC) in 26 eyes, cortical (CC) in 19 eyes and nuclear opacities (NO) in 15 eyes.

**Figure 2:**
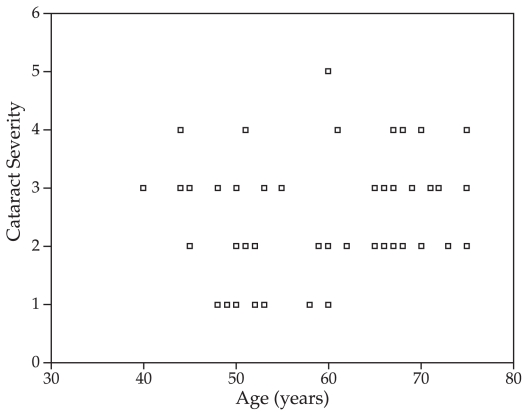
Correlation between age and cataract severity in patients with age-related cataract (rs=0.27).

Spearman correlation coefficient showed that patients with more severe opacities were older (Fig. 2; r=0.27, P=0.036). Increasing cataract severity was also correlated with decreasing VA (P<0.001, F=14.27). Contrast sensitivity values with and without glare in day and night light were compared among different types of cataract at all spatial frequencies. Contrast sensitivity was significantly reduced in all types of cataracts at all tested spatial frequencies without glare (Table 1). LSD test of significant differences between each paired group revealed that mean contrast sensitivity was significantly reduced at high spatial frequency (18 cpd) in cortical cataracts in the presence of glare in day light (P<0.001) and at low spatial frequency (3 cpd) in night light (P<0.001).

Increased cataract severity was correlated with decreased AULCSF in conditions without glare (P<0.001, F=15.39). A higher correlation was found in day light glare compared to conditions without glare (P<0.001, F=21.36). The correlation between cataract severity and AULCSF in night light glare was also significant, but less than that seen without glare (P<0.001, F=12.91).

## DISCUSSION

In this study, we evaluated the effect cataract type and severity on Snellen VA and contrast sensitivity with and without glare using the Vector Vision CSV-1000E. The fact that there was significant reduction in CS at intermediate and high spatial frequencies implied that CS testing may be more sensitive and serve as an adjunct to traditional acuity testing in quantifying the level of visual dysfunction in cataract patients.^7^ By limiting our analysis to eyes with a pure type of lens opacity, we attempted to eliminate the confounding effect of different types of coexisting opacities. The infrequent occurrence of pure PSC (only 26 eyes were available for this study) suggests that the data relating to this type of cataract must be interpreted cautiously.

Cataracts are known to increase intraocular scatter, thereby reducing retinal image contrast.^26^,^27^ Some studies suggest that contrast sensitivity should be measured at low spatial frequencies in eyes with cataract.^28^,^29^ Other studies have suggested that high spatial frequency contrast sensitivity is more informative.^30^ In the present study, we used Vector Vision CSV-1000E chart with contrast sensitivity at all spatial frequencies (low, intermediate and high). We found that increasing cataract severity is associated with a progressive decrease in both VA and AULCSF in conditions with and without glare. Decrease in AULCSF means that contrast sensitivity is decreased at all spatial frequencies on the Vector vision CSV-1000 chart. The effect of light scatter on the retinal image results in decreased retinal contrast.^15^,^18^

In a similar study with different methodology, Lasa et al^31^ found that after adjusting for visual acuity, contrast sensitivity loss was significant only in patients with advanced CC or PSC. Elliott and Gilchrist^15^ reported that eyes with early nuclear or cortical cataracts had no loss of contrast sensitivity at the lowest spatial frequency, whereas eyes with PSC had contrast sensitivity loss at low spatial frequencies. Stifter et al^32^ indicated that early PSC and CC opacities cause significant reduction in contrast sensitivity at intermediate and high spatial frequencies, but high grade cataracts reduce contrast sensitivity at all spatial frequencies. They also demonstrated that early nuclear cataracts cause no loss of contrast sensitivity at the lowest spatial frequency, whereas eyes with PSC show contrast sensitivity loss even at low spatial frequencies.

Higher-order aberrations are significantly correlated with contrast sensitivity at intermediate to high spatial frequencies in eyes with cataract. Fujikado et al^12^ found that loss of contrast sensitivity was closely related to the optical density of the cataract, which confirms our findings. However it is still unclear whether front or back light scatter, or higher-order aberrations contribute more significantly to optical degradation in eyes with cataract leading to decreased contrast sensitivity. In our study, contrast sensitivity without glare was reduced in all types of cataracts; but the difference in contrast sensitivity among the three types of cataract was not statistically significant.

In the current study, contrast sensitivity declined in the presence of glare (day and night light) with cortical cataracts. Cortical cataracts tend to form wedge-shaped spokes around the pupil that mainly affect high spatial frequencies. These opacities may cause significant glare when they encroach upon the visual axis.^31^,^32^ Although nuclear cataracts are located centrally, they are more diffuse in nature, unlike PSC opacities which are usually more dense and discrete.^32^ Measurement of CS at all spatial frequencies in the presence of glare in night or day light conditions appears to be helpful in assessment of visual function in patients with cortical and posterior subcapsular cataracts. The Vector Vision Contrast sensitivity test with glare source measures visual performance under real life conditions and gives a more complete quantification of visual function. In our study, the severity of cortical cataracts was greater than posterior subcapsular and nuclear opacities. Late cortical cataracts are associated with denser opacities in the center of the lens affecting the visual axis, therefore contrast sensitivity is more severely reduced with this type of cataracts.

Chua et al^33^ found no correlation between cataract type or severity and glare disability which is in contrast to our findings. Interestingly, Chua demonstrated that axially located cortical cataracts most significantly affected visual function in terms of VA, CS and glare disability.

Smith and Holladay^34^ have stated that the effect of a central obstruction in the aperture of a diffraction limited system is a decrease in its modulation transfer function (MTF) at low spatial frequencies. Coatings that reduce transmission at the center of the aperture tend to decrease MTF at low spatial frequencies while reduced transmission at the edge of the aperture preferentially affects high frequencies.^35^ In night light conditions and with a glare light source, miosis occurs resulting in decreased MTF at low spatial frequencies with significant cortical cataracts encroaching upon the pupil.

Williamson et al^20^ reported that all forms of cataract are associated with glare disability. They found that posterior subcapsular opacities induce more severe glare loss than cortical cataracts. In our study however, cataract severity was greatest with cortical opacities, therefore affecting glare sensitivity more than other types.

Martin et al^36^ demonstrated that regarding both VA and CS, the effect of increasing cataract severity was greatest for nuclear and smallest for cortical opacities, which is different from our findings.

Previous studies have suggested that cataracts predominantly affect CS at high spatial frequencies.^1^,^8^,^26^ We also found that contrast sensitivity is decreased at high spatial frequencies in cortical cataracts in the presence of day light glare.

The advantage of the current study is the use of AULCSF which reflects the entire sensitivity of the visual system to contrast. We also evaluated the effect of cataract type and severity on VA and CS with and without glare.

In summary, increased cataract severity, as determined by LOCS III grading, is strongly associated with a decrease in both VA and contrast sensitivity measured as AULCSF. Measurement of VA alone is insufficient for evaluating visual complaints in patients with cataracts and examinations simulating usual conditions of everyday life such as evaluation of contrast sensitivity in conjunction with a glare source may be of great utility.

## Figures and Tables

**Table 1:** Contrast sensitivity without glare and with glare in day and night light

Spatial Frequency (cpd)	Cataract Type	Contrast Sensitivity (Mean ± SD)			P-value
		Without glare	Glare day light	Glare night light	
3	PSC	0.81 ± 0.24	1.15 ± 0.41	0.45 ± 1.32	<0.001
	Cortical	0.8 ± 0.24	1.03 ± 0.38	1.25 ± 0.40	
	Nuclear	0.92 ± 0.35	1.26 ± 0.36	0.34 ± 1.46	
6	PSC	1.036 ± 0.27	1.28 ± 0.38	1.44 ± 0.45	<0.001
	Cortical	1.02 ± 0.28	1.23 ± 0.31	1.40 ± 0.40	
	Nuclear	1.1 ± 0.32	1.40 ± 0.40	1.44 ± 0.41	
12	PSC	0.69 ± 0.21	0.96 ± 0.35	1.13 ± 0.44	<0.001
	Cortical	0.71 ± 0.27	0.88 ± 0.27	1.11 ± 0.44	
	Nuclear	0.89 ± 0.32	1.16 ± 0.42	1.27 ± 0.47	
16	PSC	0.27 ± 0.27	0.44 ± 0.42	0.66 ± 0.51	<0.001
	Cortical	0.29 ± 0.26	0.38 ± 0.27	0.55 ± 0.48	
	Nuclear	0.51 ± 0.29	0.77 ± 0.40	0.95 ± 0.34	

cpd, cycle per degree; SD, standard deviation; PSC, posterior subcapsular
